# Editorial: The role of iron in cancer progression

**DOI:** 10.3389/fonc.2022.1026420

**Published:** 2022-09-21

**Authors:** Ahmed Hamaï, Chang Gong, Maryam Mehrpour

**Affiliations:** ^1^ Inserm U1151, Institut Necker Enfants Malades, Faculté de Médecine, Team 5, Université Paris Cité, Paris, France; ^2^ FEROSTEM Group, Institut Necker Enfants Malades, Inserm U1151, Université Paris Cité, Paris, France; ^3^ Guangdong Provincial Key Laboratory of Malignant Tumor Epigenetics and Gene Regulation, Sun Yat-Sen Memorial Hospital, Sun Yat-Sen University, Guangzhou, China; ^4^ Breast Tumor Center, Sun Yat-Sen Memorial Hospital, Sun Yat-Sen University, Guangzhou, China

**Keywords:** iron, cancer, ferritinophagy, ferroptosis, antitumor immunity, cancer stem cell (CSC), ferroptosis-related gene prognostic index

Iron is an essential nutrient in all mammals, involved in key biological processes, including oxygen transport, mitochondrial respiration, metabolism, detoxification, and immune defense. The ability of iron to alternate between the oxidized form and the reduced form contributes to the formation of free radicals, with an excess leading to lipid peroxidation, increased production of reactive oxygen species (ROS), oxidative stress, and DNA damage. The accumulation of iron and ROS are linked to various pathologies, including iron overload diseases and cancer. Indeed, cancer cells exhibit an increased iron demand compared to non-cancer cells. Furthermore, pathways of iron uptake, storage, mobilization, trafficking, and regulation are all perturbed in cancer, suggesting that the reprogramming of iron metabolism is a central aspect of tumor cell survival (1). Anemia is frequently observed in many patients with cancer, and iron dyshomeostasis is implicated in numerous types of cancer (2). Recent studies have shed light on the role of iron metabolism in cancer stem cells (CSC) and suggest that specific targeting of iron metabolism in CSCs may improve the efficacy of cancer therapy (3–6). This iron dependency can make CSC and non-CSC cells more vulnerable to a non-apoptotic form of regulated cell death, referred to as ferroptosis. This cell death process characterized by the iron-dependent accumulation of lipid peroxides is morphologically, biochemically, and genetically different from other well-known modalities of regulated cell death, including apoptosis, necroptosis, various forms of necrosis, and autophagy. In distinct cancer types, metabolic reprogramming has been linked to an acquired sensitivity to ferroptosis, thus opening new opportunities to treat tumors unresponsive to other therapies. The activation of ferritinophagy, a specific form of macroautophagy required for the degradation of ferritin, the main cellular iron-storage protein, seems to occur during the early initiation stage of ferroptosis (7). Recent discoveries have highlighted the importance of transferrin trafficking and ferritinophagy, as critical determinants of ferroptosis sensitivity *via* an increase in the so-called labile iron pool. Guo et al., bring us a review of the latest research about iron metabolism disorders in various types of tumors, the functions and properties of iron in ferroptosis and ferritinophagy, and new opportunities for iron-based on treatment methods for tumors, providing more information regarding the prevention and treatment of tumors. In their review, Chen et al. focus on the regulatory roles of the human six-transmembrane epithelial antigen of the prostate (STEAP) metaloxidoreductase proteins in the occurrence and development of malignant tumors. In addition, Zhang et al. summarize for us the recent findings on the role of Nuclear Factor (erythroid-derived 2)-like 2 (NRF2) as a potential modulator for orchestrating iron homeostasis and redox balance in cancer cells. Accordingly, Bajbouj et al. identified how vitamin D alters the redox balance in breast cancer cells by disrupting the cellular iron metabolism to induce oxidative stress and cell death. Zhang et al. performed us a bibliometric and knowledge-map analysis to evaluate the knowledge base, find the hotspot trends, and detect the emerging topics regarding ferroptosis research. From the FerrDb website database (the first database of ferroptosis genes and ferroptosis-diseases associations) or MSigDB and public databases, several studies presented here systematically investigated the correlation between ferroptosis-related genes and tumor patient prognosis and establish/validate a novel prognostic model of tumor patient based on ferroptosis-related gene (FRG) signature in order to develop individualized treatments and to improve their overall survival. Tian et al. validated their 7-FRG prognostic signature (including *ALOX12B*, *ALOX15*, *GPX2*, *DDIT4*, *GDF15*, *SLC2A1*, *RRM2*) in lung adenocarcinoma. For overall survival prediction in patients with breast cancer, Zhu et al. and Li et al., developed and validated a novel robust FRG panel, consisting with the 11-gene core (*CISD1*, *TP63*, *BRD4*, *PROM2*, *EMC2*, *G6PD*, *PI3KCA*, *FLT3*, *IFNG*, *ANO6*, *SLC1A4*) and the 9-gene core (*ALDH3A2*, *SIAH2*, *G6PD*, *SLC1A4*, *FLT3*, *SQLE*, *EGLN2*, *SFXN5*, *CHAC1*), respectively. In addition, He et al., identified and validated the prognostic value of a 10 FRG core signature (including *MAP1LC3A*, *SLC7A5*, *OTUB1*, *PRDX6*, *MAP3K5*, *SOCS1*, *ATG5*, *DDIT4*, *ACSL3*, *PRKAA2*) in patients with head and neck squamous cell carcinoma. Lv et al. identified 17 FRG associated with long-term of prostate cancer and finally constructed a signature based on nine FRGs (*AIFM2*, *AKR1C1*, *AKR1C*, *CBS*, *FANCD2*, *FTH1*, *G6PD*, *NFS1*, *SLC1A5*), with a prognostic value in patients with prostate cancer. If iron metabolism plays a crucial role in the occurrence and development of colon adenocarcinoma, Yuan et al., revealed the prognostic value of iron metabolism-related genes *SLC48A1* and *SLC39A8* in colon cancer. These findings provide evidence on the key role of ferroptosis in cancer development. Interestingly, most of these studies found a strong correlation between ferroptosis and immune status and immune checkpoint genes. Indeed, iron metabolism, inflammation, and immunity are tightly interlinked (8). Recent data demonstrate that immune checkpoint inhibition stimulates interferon-γ production by CD8+ T cells to kill cancer cells through the induction of ferroptosis cell death (9). In contrast, a newly developed hypothesis suggests that a ferroptosis-sensitive state may allow cancer cells to generate lipid-derived mediators modulating intra- and intercellular signaling pathways that would lead to the growth of the tumor (8). This theory suggests an inverse correlation between cellular peroxide tone and immune evasion. Thus, it is essential to explore the molecular mechanism of ferroptosis that inhibits tumor growth as well as the consequence of cancer cell death by ferroptosis, which potentially dampens antitumor immunity and promotes tumor growth. Yu et al. bring us a prognostic model in pancreatic cancer based on 6-FRGs (*CD44*, *MT1G*, *PTGS2*, *SAT1*, *TFRC*, *STEAP3*) to determine its immune landscape and underlying mechanisms. In line with this, Liu et al. also identified a prognostic signature associated with tumor immune microenvironment based on 6-FRGs (*HMOX1*, *KEAP1*, *HSBP1*, *SAT1*, *CISD1*, *GPX4*) in uterine corpus endometrial carcinoma. If long non-coding RNAs (lncRNAs) have been reported to be involved in tumorigenesis in several cancers, He et al. identified a ferroptosis-related lncRNA signature that could effectively stratify the prognosis of glioma patients with adequate predictive performance to all clinical index. Interestingly, the authors indicated that there was increased immune infiltration in the high-risk group defined by the ferroptosis-related lncRNA signature. Accordingly, Duan et al., explored the prognostic value of a novel comprehensive biomarker, the iron-monocyte-to-lymphocyte ratio or IronMLR score, in patients with early-stage triple-negative breast cancer, illustrating that the iron-inflammation axis might be a potential prognostic biomarker of survival outcomes. Hua et al. and Duan et al. also explored a novel prognostic model based on the serum metal levels, iron and copper, respectively, for patients with early-stage triple-negative breast cancer as a practical tool for individualized survival predictions and treatment guidance.

Overall, we believe that this Research Topic opens up new and exciting fields of investigation into iron metabolism in cancer progression and in cancer treatments, and hope readers will enjoy it.

## Author contributions

All authors listed have made a substantial, direct, and intellectual contribution to the work and approved it for publication.

## Funding

The authors gratefully acknowledge funding from INSERM, Université Paris Cité, la ligue nationale contre le cancer, and Comité de Paris de la ligue contre le cancer.

## Conflict of interest

The authors declare that the research was conducted in the absence of any commercial or financial relationships that could be construed as a potential conflict of interest.

## Publisher’s note

All claims expressed in this article are solely those of the authors and do not necessarily represent those of their affiliated organizations, or those of the publisher, the editors and the reviewers. Any product that may be evaluated in this article, or claim that may be made by its manufacturer, is not guaranteed or endorsed by the publisher.
